# Supplementary material for: RANKL Inhibition Reduces Cardiac Hypertrophy in mdx Mice and Possibly in Children with Duchenne Muscular Dystrophy
Source: Cells. 2023 Jun 3;12(11):1538. doi: 10.3390/cells12111538 (PMC10253225; doi:10.3390/cells12111538)
Supplement: Supplementary file 1 [file cells-12-01538-s001.zip › cells-2404453-supplementary.pdf]

SUPPLEMENTAL MATERIAL

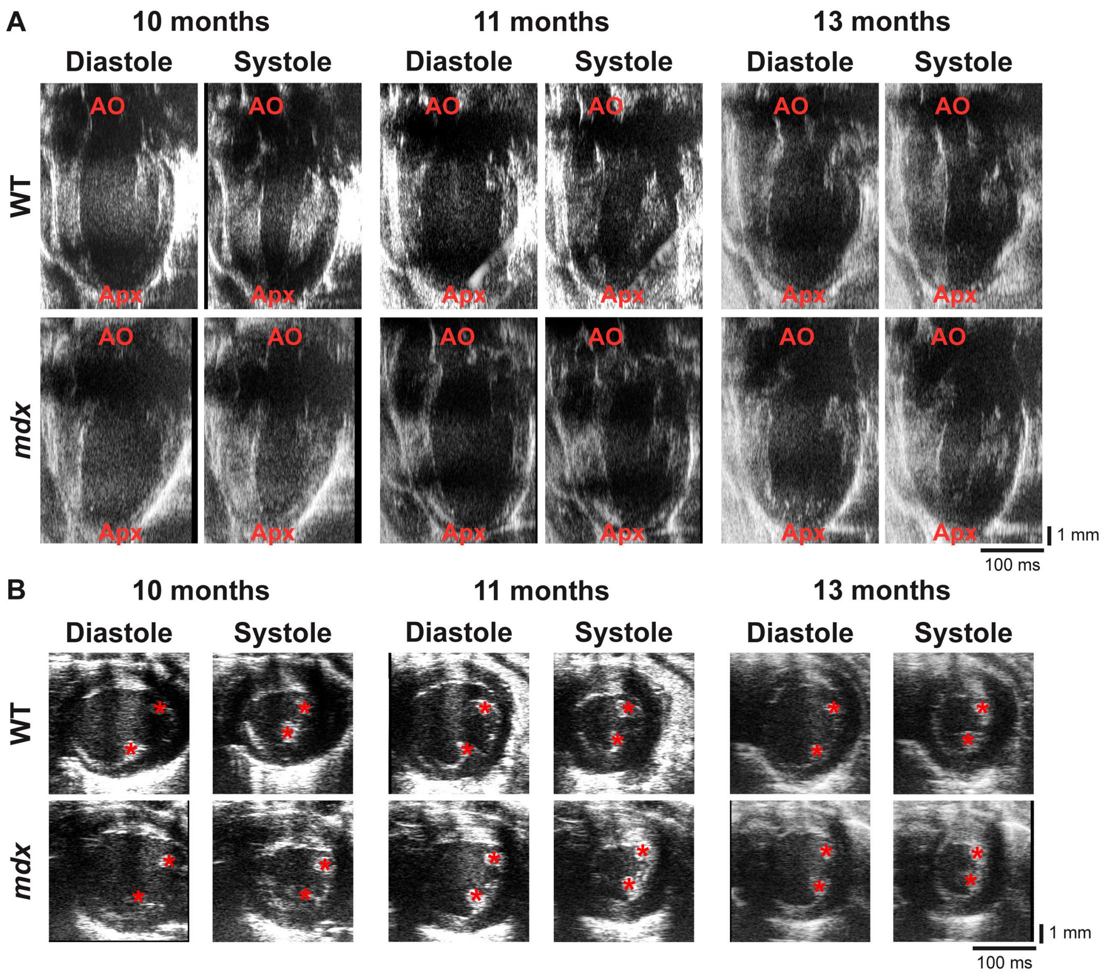

Supplemental Figure S1. Representative echocardiography B-mode images of hearts from WT and *mdx* heart mice at 10, 11, and 13 months of age. Para-sternal long-axis (A) and short-axis (B) of the left ventricle. Vertical and horizontal scale bars represent the size in mm and the time in ms, respectively. AO, aorta; Apx, apex (A). The red asterisks indicate papillary muscles (B).

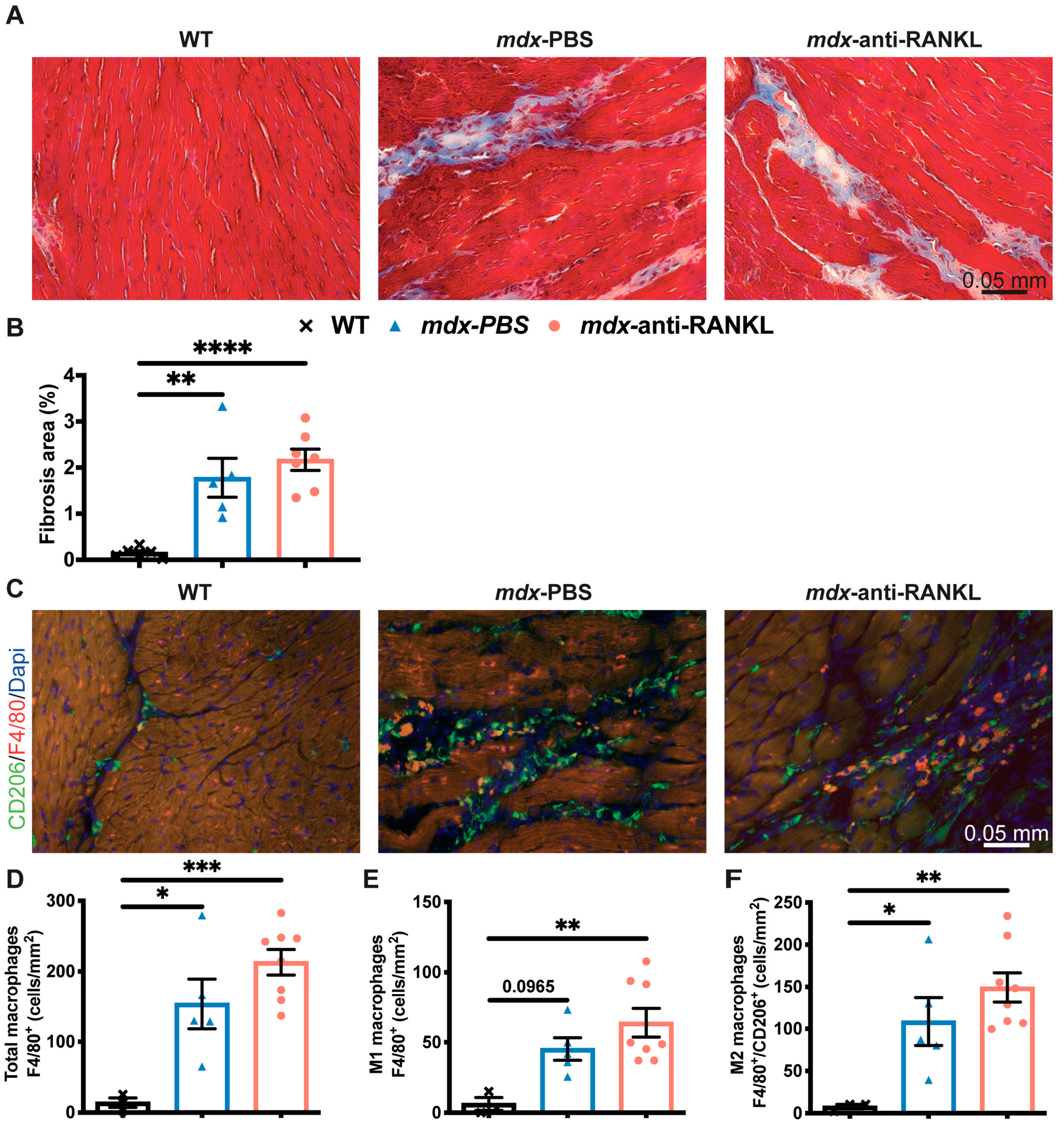

Supplemental Figure S2. Representative Masson's trichrome staining of cardiac tissue (A). Fibrosis area quantification (B). We examined inflammation by labelling heart sections with F4/80 (red), a marker for all macrophage phenotypes, and CD206 (green), an M2-macrophage specific antibody, and DAPI (blue) for nuclei (C). Count of the total number of macrophages (D), M1-macrophages (E) and M2-macrophages (F). Data are presented as means ± SEM.*p<0.05, **p<0.01 and ***p<0.001, ****p<0.0001, determined by unpaired two-tailed Student's t-test. The scale bar in A and C represents 0.05 mm. n=6 for WT, n=5 for *mdx* PBS and n=7 for *mdx* anti-RANKL for fibrosis (A, B) and n=3 for WT, n=5 for *mdx* PBS and n=8 for *mdx* anti-RANKL for macrophages (C-F).

Table S1. Cardiac parameters measured by echocardiography in WT and *mdx* mice at 10, 11 and 13 months of age

Age	10 months		11 months		13 months	
Mouse strain	WT (n=9)	*mdx* (n=13)	WT (n=11)	*mdx* (n=11)	WT (n=11)	*mdx* (n=12)
LVAW;d (mm)	0.81 ± 0.04	1.13 ± 0.05 ***	1.07 ± 0.08	1.26 ± 0.07	1.33 ± 0.07 ††††	1.44 ± 0.06 †††
LVAW;s (mm)	1.41 ± 0.08	1.66 ± 0.07 *	1.66 ± 0.09	1.74 ± 0.09	1.81 ± 0.08 ††	1.81 ± 0.06
LVPW;d (mm)	0.73 ± 0.05	0.84 ± 0.04	0.87 ± 0.04	0.94 ± 0.06	0.97 ± 0.07 †	1.04 ± 0.06 †
LVPW;s (mm)	1.16 ± 0.09	1.27 ± 0.04	1.46 ± 0.06	1.34 ± 0.08	1.40 ± 0.09	1.42 ± 0.08
LVID;d (mm)	4.22 ± 0.04	3.69 ± 0.1 ***	3.78 ± 0.1	3.69 ± 0.1	3.51 ± 0.2 ††	3.67 ± 0.09
LVID;s (mm)	2.72 ± 0.1	2.31 ± 0.1 *	2.19 ± 0.2	2.44 ± 0.2	2.20 ± 0.2	2.68 ± 0.1
Volume;d (µL)	79.8 ± 1.88	58.7 ± 3.62 ***	62.6 ± 5.43	59.9 ± 6.60	53.7 ± 6.72 ††	57.6 ± 3.24
Volume;s (µL)	28.3 ± 3.01	19.1 ± 1.90 *	18 ± 3.35	23.5 ± 4.41	20.5 ± 4.49	27.7 ± 3.13
Stroke Volume (µL)	51.5 ± 2.48	39.7 ± 2.05 **	44.7 ± 2.54	36.3 ± 2.76 *	33.2 ± 2.83 ††††	29.9 ± 1.33 ††
EF (%)	64.8 ± 3.46	68.2 ± 1.59	73.5 ± 2.97	63.8 ± 3.78	66.5 ± 4.30	53.3 ± 3.34 * ††
FS (%)	35.8 ± 2.65	37.6 ± 1.24	42.9 ± 2.73	34.9 ± 2.92	37.1 ± 3.27	27.4 ± 2.30 * †
Heart Rate (bpm)	459 ± 45	448 ± 16.5	507 ± 17.2	479 ± 26.7	481 ± 16.5	480 ± 12.6
Cardiac Output (mL/min)	24.0 ± 2.82	17.7 ± 1.12 *	22.3 ± 0.9	16.9 ± 1 ***	15.8 ± 1.2 ††	14.3 ± 0.7
LV mass (mg)	99.2 ± 6.93	112 ± 6.63	113 ± 4.51	128 ± 4.84	128 ± 5.16 †	155 ± 5.82 ††††

LVAW : Left ventricular anterior wall thickness end diastole (d) and end systole (s). LVPW : Left ventricular posterior wall thickness end diastole (d) and end systole (s). LVID : Left ventricular internal diameter end diastole (d) and end systole (s). EF : Ejection fraction. FS : Fractional shortening. Data are expressed as means ± SEM. P values for significant differences between WT and *mdx* values at a given age are represented by: *p<0.05, **p<0.01, ***p<0.001, or ****p<0.0001. P values for significant differences between 10 and 13 months within strain WT or *mdx* are represented by: †p<0.05, ††p<0.01, †††p<0.001, or ††††p<0.0001.

Table S2. Cardiac parameters measured by echocardiography in *mdx* mice before and after treatment, at 11 and 13 months of age, respectively

Mouse strain and treatment	*mdx*-PBS (n=19)		*mdx*-anti-RANKL (n=16)	
Age (month)	11 pre-treatment	13 post-treatment	11 pre-treatment	13 post-treatment
LVAW;d (mm)	1.19 ± 0.05	1.36 ± 0.05 *	1.25 ± 0.04	1.10 ± 0.04 * †††
LVAW;s (mm)	1.70 ± 0.08	1.75 ± 0.06	3.67 ± 0.1	3.43 ± 0.09
LVPW;d (mm)	0.966 ± 0.04	1.03 ± 0.04	0.989 ± 0.05	0.932 ± 0.03
LVPW;s (mm)	1.37 ± 0.06	1.39 ± 0.06	1.34 ± 0.08	1.32 ± 0.05
LVID;d (mm)	3.59 ± 0.1	3.56 ± 0.09	3.67 ± 0.1	3.43 ± 0.09
LVID;s (mm)	2.33 ±0.2	2.54 ± 0.1	2.51 ± 0.2	2.21 ± 0.1
Volume;d (µL)	56.6 ± 5.29	54 ± 3.30	59 ± 4.24	49.3 ± 3.27
Volume;s (µL)	22.3 ± 3.79	24.8 ± 2.58	25.4 ± 3.19	18.2 ± 2.71
Stroke Volume (µL)	34.3 ± 2.16	29.2 ± 1.36	33.6 ± 2.01	31.1 ± 2.01
EF (%)	65.4 ± 3.54	56 ± 2.43 *	60.4 ± 3.80	64.9 ± 3.81 †
FS (%)	36.6 ± 2.73	29 ± 1.62 *	33.1 ± 3.20	36 ± 2.89 †
Heart Rate (bpm)	483 ± 22.9	479 ± 11.5	451 ± 16.3	498 ± 14 *
Cardiac Output (mL/min)	16.1 ± 0.9	13.9 ± 0.6	15 ± 1	15.4 ± 0.9
LV mass (mg)	121 ± 6.23	140 ± 5.98 *	132 ± 5.56	104 ± 4.61 *** ††††

LVAW : Left ventricular anterior wall thickness end diastole (d) and end systole (s). LVPW : Left ventricular posterior wall thickness end diastole (d) and end systole (s). LVID : Left ventricular internal diameter end diastole (d) and end systole (s). EF : Ejection fraction. FS : Fractional shortening. Data are expressed as means ± SEM. P values for significant changes overtime within treatment groups are represented by: *p<0.05, **p<0.01, ***p<0.001, or ****p<0.0001. P values for significant differences between treatment groups are represented by: †p<0.05, ††p<0.01, †††p<0.001, or ††††p<0.0001.
